# Editorial: Mechanotransduction in cancer progression and immune response

**DOI:** 10.3389/fcell.2026.2032624

**Published:** 2026-10-30

**Authors:** Sangyoon Joshua Han

**Affiliations:** Department of Biomedical Engineering, Michigan Technological University, Houghton, MI, United States

**Keywords:** biomechanics, cancer metastasis, lymph node, matrix stiffness, mechanotransduction, tumor microenvironment, tumor-associated macrophages

## Introduction

Tumors are not only biochemical environments. As they grow, they stiffen, compress their vasculature, build pressure gradients, and reorganize collagen into aligned tracks, and every cell in this landscape, whether malignant, stromal, or immune, must interpret these physical cues. Mechanotransduction, the conversion of mechanical input into biochemical signaling, has therefore emerged as a regulator of invasion, metastasis, immune surveillance, and therapeutic response. This Research Topic brought together five articles on how the mechanical properties of the tumor microenvironment shape cancer progression and immune behavior, with particular interest in immune cell migration and infiltration as a function of tissue stiffness. Together they suggest that the field is moving from cataloguing mechanosensitive molecules toward asking where, when, and in which cells mechanical cues are decisive.

## Mechanics reaching the immune compartment

Two reviews examine how physical cues are read by immune cells and immune organs. Kim et al. focus on tumor-associated macrophages (TAMs), organizing the literature around cellular origin, mechanical regulation, and experimental models. They contrast monocyte-derived and tissue-resident TAMs, then ask how a dense, stiff matrix affects TAM infiltration, polarization, and spatial organization. A notable message is that macrophages may exploit fibrotic matrix rather than simply being excluded by it: stiffness-dependent podosome-like structures and changes in migration mode can support movement through dense collagen, while the same mechanical environment shapes polarization and tumor-supportive functions. The authors also compare hydrogel, spheroid and organoid, and microfluidic platforms, and note that none yet captures the heterogeneity of TAM states.

Chen et al. turn to the lymph node, reframing tumor-draining lymph nodes from passive relay stations into mechanically and immunologically active organs. They connect solid stress, matrix stiffness, and interstitial fluid pressure to lymphatic drainage and to the formation of immunosuppressive premetastatic niches, and use this framework to evaluate lymph node de-escalation in breast cancer, melanoma, head and neck, lung, and gastrointestinal cancers. Their proposal of selective preservation and selective intensification, guided by functional rather than purely anatomical criteria, shows how mechanobiology can inform surgical decision making. Importantly, the spatial mechanics and immune fate hypothesis they put forward is presented as testable rather than established.

## Lung cancer across scales

Two reviews address lung cancer at different scales. Guo et al. focus on fibrosis-associated disease, where matrix stiffening and Piezo1-dependent signaling are proposed to form a mechano-immunological barrier, and where normalization of the microenvironment is considered a therapeutic aim. Wang et al. look inside the cell. They propose a mechanochemical coupling framework in which high-magnitude cyclic stretch and matrix stiffening, acting through Piezo1 and integrin-Rac1 signaling, are hypothesized to drive oxidative stress, mitochondrial fragmentation, altered nuclear mechanics, and impaired DNA repair, with consequences for genomic instability, epithelial-mesenchymal transition, inflammasome activation, and immune evasion. The review presents this cascade explicitly as a hypothesis and offers mechanopharmacology as a theoretical strategy, which helps readers separate what is proposed from what is established.

## From mechanics to clinical prediction

Zhang et al. contribute the original research article of the Research Topic. Using transcriptomic data from GTEx, TCGA, and GEO, they identify tumor mechanics-related genes in cervical cancer and build a seven-gene prognostic model that stratifies patients into risk groups differing in mutational landscape, immune infiltration, and chemotherapy response. In a clinical cohort, they further report a moderate positive correlation between tumor stiffness measured by strain elastography and MMP1 expression, an association the authors appropriately describe as preliminary. This work illustrates one route by which mechanobiological concepts can be operationalized with genomic and clinical data, and it links a physical measurement, tissue stiffness, to a molecular readout.

## Common themes and open questions

Several themes recur across these articles. First, the authors distinguish plausibility from causality. Many mechanisms rest on *in vitro* systems or on tissues and cell types other than the tumor in question, and the contributors are explicit about where direct *in vivo* or patient evidence is missing. Second, separating mechanical from biochemical cues remains difficult. Stiffness, density, pore size, and confinement covary in common hydrogels, and architecture and soluble factors are entangled in spheroids and organoids. Third, context dependence is the rule: the same force can be protective or pathogenic depending on cell type, timing, and tissue architecture. Fourth, immune populations are heterogeneous, and current models rarely reproduce that diversity. Finally, translation will depend on measurable physical biomarkers and on interventions that modify mechanics locally rather than through broad systemic inhibition.

Although this Research Topic encouraged studies of immune cell migration under varying stiffness, the contributions concentrate on macrophages and lymph nodes. Migration of T cells and natural killer cells through stiff, confined matrices, and the use of live-cell imaging, force microscopy, and bioMEMS to measure it in intact tissue, remain open opportunities. We hope this Research Topic encourages work that links molecular mechanosensing to immune function at the tissue scale and, ultimately, to patient outcomes.

We thank all authors and reviewers for their contributions to this Research Topic.

